# Efficacy of Procyanidins against In Vivo Cellular Oxidative Damage: A Systematic Review and Meta-Analysis

**DOI:** 10.1371/journal.pone.0139455

**Published:** 2015-10-01

**Authors:** Shugang Li, Mengchuan Xu, Qiang Niu, Shangzhi Xu, Yusong Ding, Yizhong Yan, Shuxia Guo, Feng Li

**Affiliations:** 1 Department of Public Health and Key Laboratory of Xinjiang Endemic and Ethnic Diseases (Ministry of Education), Shihezi University School of Medicine, Xinjiang, China; 2 Department of Pathology and Key Laboratory of Xinjiang Endemic and Ethnic Diseases (Ministry of Education), Shihezi University School of Medicine, Shihezi, Xinjiang, 832002, China; University of Catania, ITALY

## Abstract

**Aims:**

In this study, the efficacy of proanthocyanidins (PCs) against oxidative damage was systematically reviewed to facilitate their use in various applications.

**Methods:**

A meta-analysis was performed by two researchers. Each investigator independently searched electronic databases, including Cochrane, PubMed, Springer, Web of Science, China National Knowledge Infrastructure (CKNI), China Science and Technology Journal Database (CSTJ), and WanFang Data, and analyzed published data from 29 studies on the effects of PCs against oxidative damage. Oxidative stress indexes included superoxide dismutase (SOD), malondialdehyde (MDA), catalase (CAT), glutathione (GSH), glutathione peroxidase (GPx), and total antioxidative capacity (T-AOC).

**Results:**

Compared with the oxidative damage model group, PCs effectively improved the T-AOC, SOD, GSH, GPx, and CAT levels, and reduced the MDA levels; these differences were statistically significant (*P* < 0.05). In studies that used the gavage method, SOD (95% CI, 2.33–4.00) and GPx (95% CI, 2.10–4.05) were 3.16-fold and 3.08-fold higher in the PC group than in the control group, respectively. In studies that used the feeding method, SOD (95% CI, 0.32–1.74) and GPx (95% CI, -0.31 to 1.65) were 1.03-fold and 0.67-fold higher in the PC group than in the control group, respectively. Statistically significant differences in the effects of PCs (*P* < 0.00001) were observed between these two methods. MDA estimated from tissue samples (95% CI, -5.82 to -2.60) was 4.32-fold lower in the PC group than in the control group. In contrast, MDA estimated using serum samples (95% CI, -4.07 to -2.06) was 3.06-fold lower in the PC group than in the control group. The effect of PCs on MDA was significantly greater in tissue samples than in serum samples (*P* = 0.02).

**Conclusion:**

PCs effectively antagonize oxidative damage and enhance antioxidant capacity. The antagonistic effect may be related to intervention time, intervention method, and the source from which the indexes are estimated.

## Introduction

Oxidative stress is caused by an imbalance between the production of reactive oxygen species (ROS) and the ability of a biological system to eliminate ROS or repair the resulting damage [1]. Thus, oxidative stress may result in an increased number of free radicals and cause lipid peroxidation, eventually leading to apoptosis and many diseases [2]. Increasing evidence has shown that oxidative stress plays a particularly important role in the development of cardiovascular diseases such as atherosclerosis, hypertension, atrial fibrillation, and cardiomyopathy [3]. Many reactive substances, such as arsenic [4] and hydrogen peroxide (H_2_O_2_) [5], can result in organismal damage via ROS and oxidative stress. Therefore, it is important to repair damage using antioxidant agents.

The effects of antioxidant substances such as vitamin C [6], E [7], and luteins [8] have been extensively studied owing to their health benefits. Additionally, the relative antioxidant efficacy of these substances has been previously examined. In particular, proanthocyanidins (PCs) have gained recent attention. These polyphenols are abundant in grape, haw, and gingko [9]. PCs have high antioxidant capacities and are efficient free radical scavengers. They are highly water soluble, easy to extract, rich in various plants, and can be absorbed naturally [10]. The antioxidative effects of PCs have not been systematically reviewed; additionally, the reported antioxidant efficacy of these compounds differs among studies [11–13], and their antioxidative ability is still unclear. Therefore, we performed a systematic review and meta-analysis based on a literature search to comprehensively analyze relevant data regarding the efficacy of PCs against oxidative damage. This work provides a scientific basis for the development and utilization of PC-based resources. According to the PICOS framework, the subjects, intervention, controls, and outcomes considered in this analysis were mice, PCs, an oxidative damage model, and enzyme levels with respect to oxidative stress, respectively. Randomized controlled mouse experiments were considered.

## Materials and Methods

### Eligibility criteria

The eligibility criteria were as follows. Randomized controlled mouse experiments and studies published in either Chinese or English were included. All strains and mouse genders were included in the present study. Oxidative damage model groups induced by any substance were used as the controls. The experimental groups included interventions with PCs only. If various doses of PCs were used in a study, the highest dose was chosen for this analysis. Valid outcome measures included the levels of enzymes related to oxidative stress measured by a microplate reader. These indicators of oxidative stress included superoxide dismutase (SOD), malondialdehyde (MDA), catalase (CAT), glutathione (GSH), glutathione peroxidase (GPx), and total antioxidative capacity (T-AOC).

### Exclusion criteria

The exclusion criteria were as follows: (1) repeat publications, (2) incomplete information, (3) insufficient or insignificant statistical data, (4) unrelated to the study objectives, (5) lack of appropriate controls, and (6) reviews.

### Search strategy

Searches were performed using the electronic databases Cochrane, PubMed, Springer, Web of Science, China Science and Technology Journal Database (CSTJ), WanFang Data, and China National Knowledge Infrastructure (CKNI) (last search updated on April 30, 2015) using PICOS. The key search string was (mice OR rat) AND (procyanidins OR proanthocyanidins) AND (antioxygenation OR antioxidant OR antioxidation) and the language was restricted to English and Chinese. We read both of the title and abstract first to make a decision whether the study is suitable for our study.

### Data extraction

Two reviewers (SGL and MCX) independently screened full-length articles. The following information was extracted from the complete manuscripts of each qualified study: publication characteristics (title of the study, first author, publication date, and journal/magazine), basal data (n, mean ± SD) for the experimental and control groups, PC intervention modes, period of PC treatment, outcome indicators, and the source of indicator estimates (i.e., serum or tissue samples) (S1 File). If the two reviewers hold different opinions, then we invited the Prof. GSX, who is teaching meta-analysis subject in university, to make a final decision of the results.

### Data analysis

The mean values for each outcome indicator differed between the experimental and control groups. Significant heterogeneity was detected (*P* < 0.05, *I* ^*2*^ > 75%); therefore, a random-effects model was applied for the meta-analysis. A multivariate meta-regression analysis was performed to determine the source of heterogeneity. Continuous variables were estimated as standardized mean differences (SMDs) with 95% confidence intervals (CI) between the PC-treated animals and control animals. All reported *P*-values are two-sided and a significance level of 0.05 was used. For additional insight, subgroup analyses were performed based on intervention mode (feed or gavage), length of PC treatment (<30 d or ≥30 d), and sample source (serum or tissue samples) to determine the factors associated with differences among study results in the outcome indicators. Publication bias was explored using funnel plots. All analyses were implemented in Review Manager Version 5.2 (The Nordic Cochrane Centre, The Cochrane Collaboration, 2012) and Stata 12.0.

## Results

### Study characteristics

**Fig 1:**
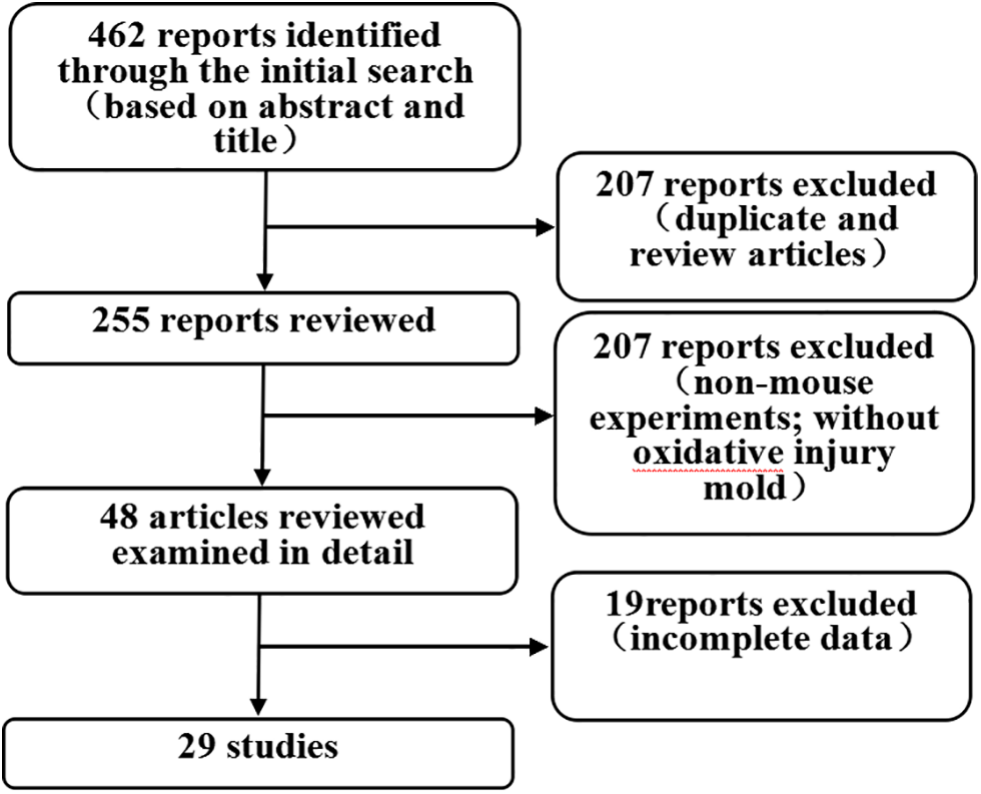
Flowchart of search strategy. The meta-analysis included animal studies that investigated the antioxidant effect of proanthocyanidins (PCs).

Using the search strategy, 462 articles were identified (Fig 1), of which 29 were valid for the meta-analysis according to the eligibility and exclusion criterias. [14–42] (Table 1). Mice were used as animal models in these studies, and each study investigated the effect of PCs on oxidative damage. The oxidative damage models were primarily mice induced by various substances (e.g., arsenite, H_2_O_2_, and fluorine), and the antioxidative damage models were provided various PCs as interventions. PCs were administered by feeding (n = 3) or gavage (n = 26). The PC intervention time varied among studies, and was categorized as <30 d (n = 17) or ≥30 d (n = 12). Oxidative stress indexes (i.e., MDA, SOD, GPx, T-AOC, GSH, and CAT) were examined using serum (n = 19) and tissue samples (n = 10).

**Table 1:** Characteristics of the animal studies included in the meta-analysis.

First author (year)	Language	n	Mode of intervention	Period of PC(day)	Source of indicators	Outcome indicators
Su-Jin2009 [14]	English	10	Feed	<30d	Tissue	2.3.6
Mi-Ok Shin2010 [15]	English	6	Feed	<30d	Tissue	1
Xiayuan2010 [16]	Chinese	8	Gavage	<30d	Serum	1.2
Adem Guler2011 [17]	English	8	Gavage	<30d	Tissue	1.2.3.4
HUANG Qi-liang2011 [18]	English	10	Gavage	≥30d	Serum	1.2
Lijianling2011 [19]	Chinese	10	Gavage	<30d	Tissue	1.2
Osama M.Ashour2011 [20]	English	12	Gavage	<30d	Tissue	1.2.5.6
Xielimei2011 [21]	Chinese	10	Gavage	≥30d	Tissue	1.2
Soo-Kyong Choi2012 [22]	English	8	Feed	<30d	Tissue	2.3.5
Vijayakumar2012 [23]	English	6	Gavage	<30d	Tissue	2.3.5.6
Wangweifen2012 [24]	Chinese	10	Gavage	<30d	Tissue	1.2
Xiao GENG2012 [25]	English	8	Gavage	<30d	Tissue	1.2
Yu Deng2012 [26]	English	8	Gavage	<30d	Tissue	1.2.3.5
zhangxuan2012 [27]	English	16	Gavage	<30d	Tissue	1.2.3.6
zhaopeng2012 [28]	Chinese	12	Gavage	≥30d	Serum	1.2.3
Bailijun2013 [29]	Chinese	5	Gavage	<30d	Serum	1.2.3.6
Dingyusong2013 [30]	Chinese	10	Gavage	≥30d	Tissue	1.2
Hanaa A2013 [31]	English	6	Gavage	≥30d	Tissue	1.2.3.4
Jiangyanfei2013 [32]	Chinese	12	Gavage	≥30d	Serum	1.2
Miaozhiru2013 [33]	Chinese	10	Gavage	≥30d	Serum	1.2.3.5
E Bakar2014 [34]	English	7	Gavage	<30d	Tissue	1.2.4.5
Gaolu2014 [35]	Chinese	10	Gavage	≥30d	Serum	1.2.3.6
Hua Zhang2014 [36]	English	10	Gavage	≥30d	Tissue	1.2.5.6
Juan Xiao2014 [37]	English	10	Gavage	<30d	Serum	1.2.3
Noorah2014 [38]	English	10	Gavage	<30d	Tissue	2.6
Tingting Ren2014 [39]	English	8	Gavage	≥30d	Serum	2.5
Wangcheng2014 [40]	Chinese	10	Gavage	≥30d	Tissue	1.2.4.5
Ying GAO2014 [41]	English	10	Gavage	≥30d	Tissue	1.2.3
Esrafil Mansouri2015 [42]	English	10	Gavage	<30d	Serum	1.2.3.6

Note: n = number of experimental animals; 1 = malondialdehyde, 2 = superoxide dismutase, 3 = glutathione peroxidase, 4 = total antioxidative capacity, 5 = glutathione, and 6 = catalase.

### Meta-analyses

#### Effect of PC on SOD

**Fig 2:**
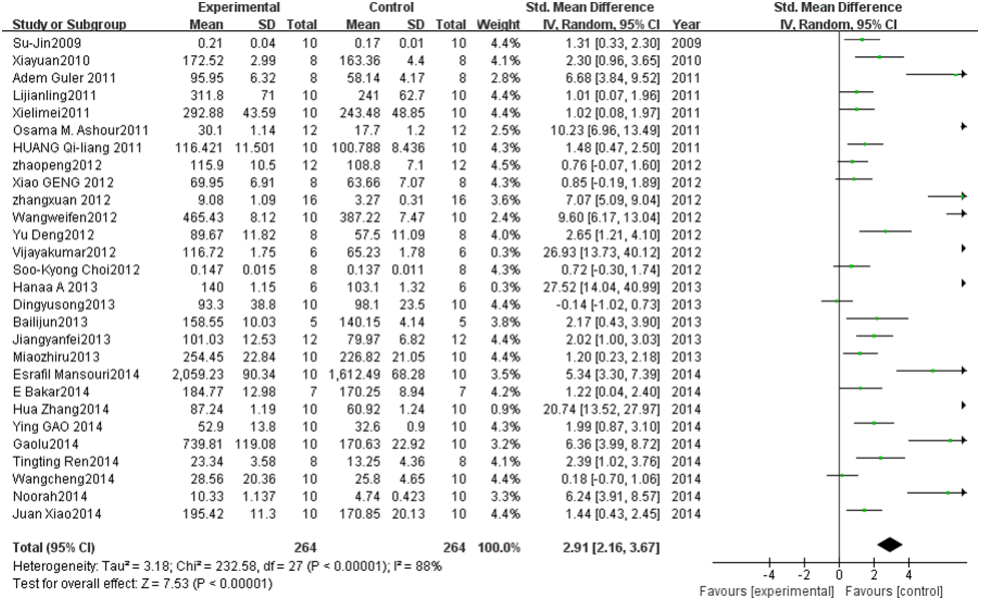
Effect of PC on superoxide dismutase (SOD). Forest plot showing the impact of PC treatment on SOD compared with controls. Abbreviations: SMD = standardized mean difference, IV = independent variable, 95% CI = 95% confidence interval.

A total of 28 studies estimated SOD levels. A pooled analysis showed that the SOD level was 2.91-fold higher in the experimental group than in the control group (95% CI, 2.16–3.67; *Z* = 7.53; *P* < 0.00001) with significant heterogeneity (*P* < 0.0001; *I* ^2^ = 88%; Fig 2).

#### Effect of PC on T-AOC

**Fig 3:**
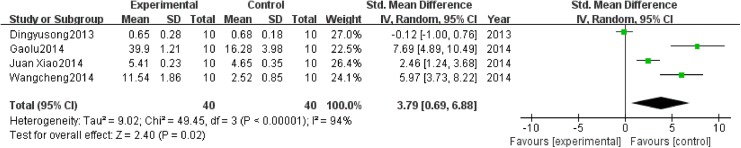
Effect of PC on total antioxidative capacity (T-AOC). Forest plot showing the impact of PC treatment on T-AOC, compared with controls. Abbreviations: SMD = standardized mean difference, IV = independent variable, 95% CI = 95% confidence interval.

A total of 4 studies estimated T-AOC levels. A pooled analysis showed that the T-AOC level was 3.79-fold higher in the experimental group than in the control group (95% CI, 0.69–6.88; *Z* = 2.40; *P* = 0.02) with significant heterogeneity (*P* < 0.0001; *I* ^2^ = 94%; Fig 3).

#### Effect of PC on GSH

**Fig 4:**
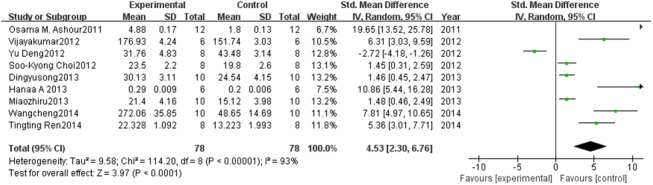
Effect of PC on glutathione (GSH). Forest plot showing the impact of PC treatment on GSH, compared with controls. Abbreviations: SMD = standardized mean difference, IV = independent variable, 95% CI = 95% confidence interval.

A total of 9 studies estimated GSH levels. A pooled analysis showed that the GSH level was 4.53-fold higher in the experimental group than in the control group (95% CI, 2.30–6.76; *Z* = 3.97; *P* < 0.0001) with significant heterogeneity (*P* < 0.00001; *I* ^2^ = 93%; Fig 4).

#### Effect of PC on GPx

**Fig 5:**
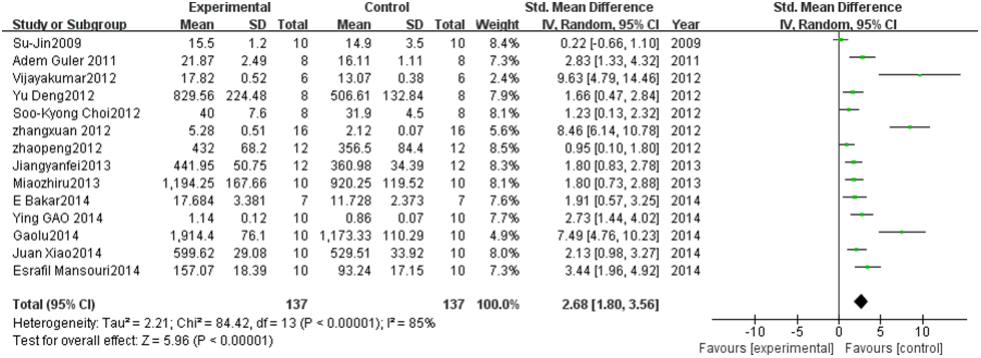
Effect of PC on glutathione peroxidase (GPx). Forest plot showing the impact of PC treatment on GPx, compared with controls. Abbreviations: SMD = standardized mean difference, IV = independent variable, 95% CI = 95% confidence interval.

Fourteen studies described GPx levels. A pooled analysis showed that the GPx level was 2.68-fold higher in the experimental group than in the control group (95% CI, 1.80–3.56; *Z* = 5.96; *P*< 0.00001) with significant heterogeneity (*P* < 0.00001; *I* ^2^ = 85%; Fig 5).

#### Effect of PC on CAT

**Fig 6:**
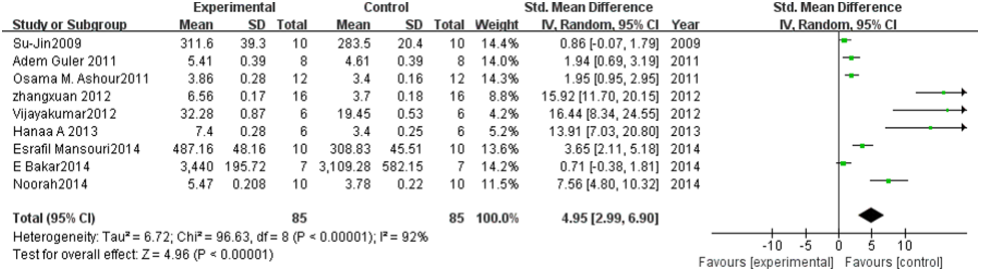
Effect of PC on catalase (CAT). Forest plot showing the impact of PC treatment on CAT, compared with controls. Abbreviations: SMD = standardized mean difference, IV = independent variable, 95% CI = 95% confidence interval.

Nine studies estimated CAT levels. A pooled analysis showed that the CAT level was 4.95-fold higher in the experimental group than in the control group (95% CI, 2.99–6.90; *Z* = 4.96; *P* < 0.00001) with significant heterogeneity (*P* < 0.00001; *I* ^2^ = 92%; Fig 6).

#### Effect of PC on MDA

**Fig 7:**
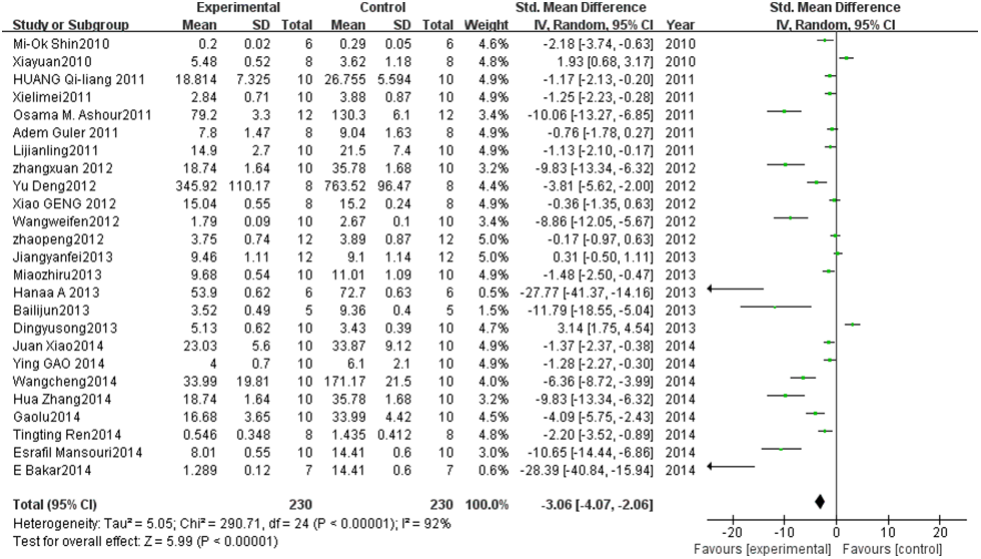
Effect of PC on malondialdehyde (MDA). Forest plot showing the impact of PC treatment on MDA, compared with controls. Abbreviations: SMD = standardized mean difference, IV = independent variable, 95% CI = 95% confidence interval.

Twenty-five studies described MDA levels. A pooled analysis showed that the MDA level was 3.06-fold lower in the experimental group than in the control group (95% CI, 4.07–2.06; *Z* = 5.99; *P* < 0.00001) with significant heterogeneity (*P* < 0.00001; *I* ^2^ = 92%; Fig 7).

#### Subgroup analyses

**Fig 8:**
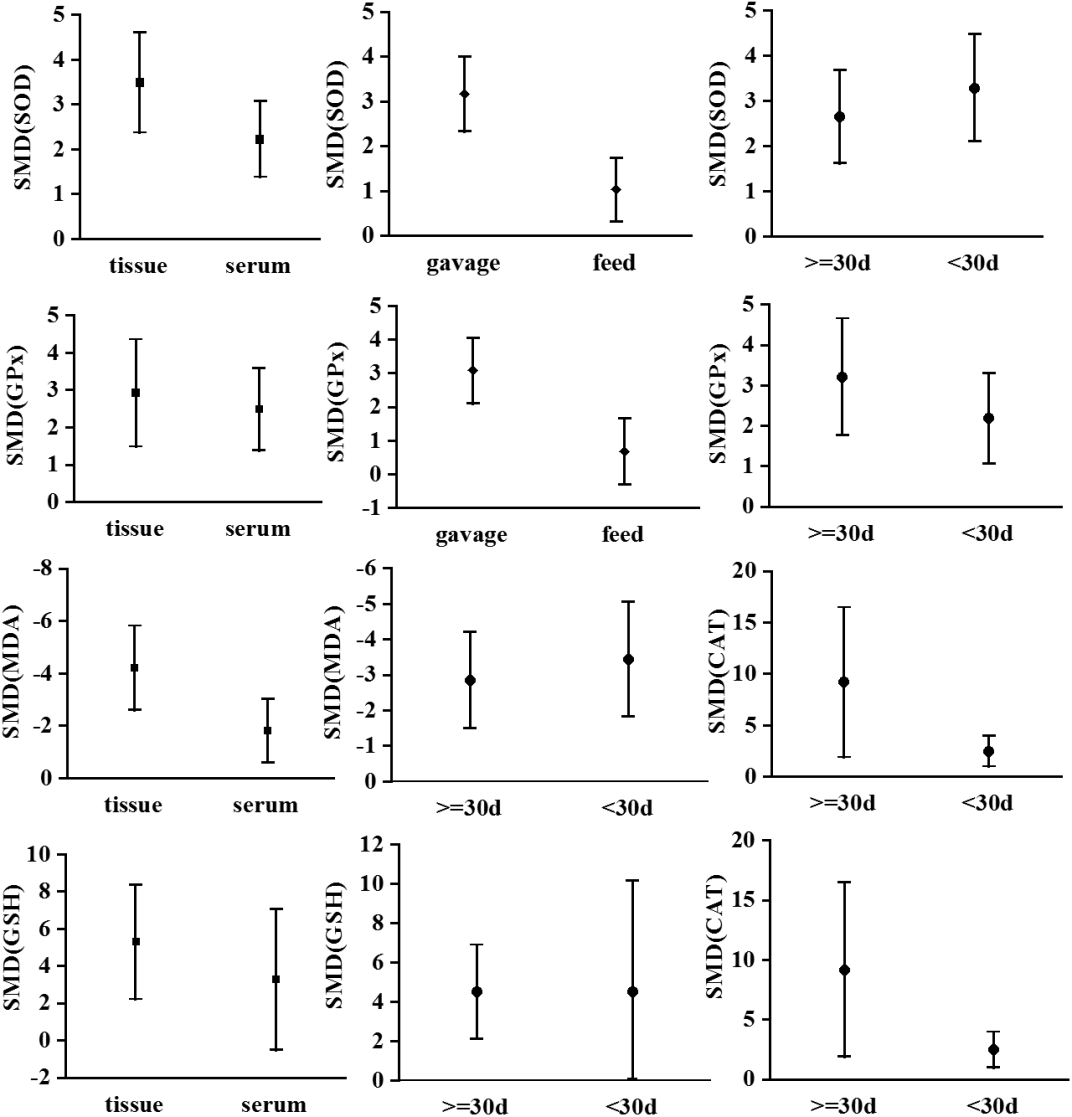
Subgroup analyses to determine the effect of PC on oxidative damage. Based on a subgroup analysis, the effect of PC using the gavage mode was stronger than that observed using the feeding mode (*P* < 0.00001; A2, B2). The effect of PC on MDA measured in tissue samples was significantly stronger than that measured in serum samples (*P* = 0.02; C1). Abbreviations: SMD = standardized mean difference.

We conducted a subgroup analysis considering the mode of intervention (gavage vs. feed), intervention period (<30 d vs. ≥30 d), and source of samples (tissue vs. serum). The SMD between PCs and control groups for SOD and GPx of tissue samples, gavage, and ≥30-d interventions were higher than those for serum samples, feeding, and <30-d interventions (*P* < 0.05, see Fig 8A1, 8A2, 8B1, 8B2 and 8B3). Furthermore, the SMD of MDA between the PC and control groups was significantly higher for tissue samples than for serum samples (*P* < 0.05, see Fig 8C1). The SMD of CAT between the PC and control groups was also higher for interventions of ≥30 d than for those of <30 d (*P* < 0.05, see Fig 8D3). We did not detect statistically significant differences in GSH or T-AOC (see Fig 8C3, 8D1 and 8D2).

#### Sensitivity analysis

**Fig 9:**
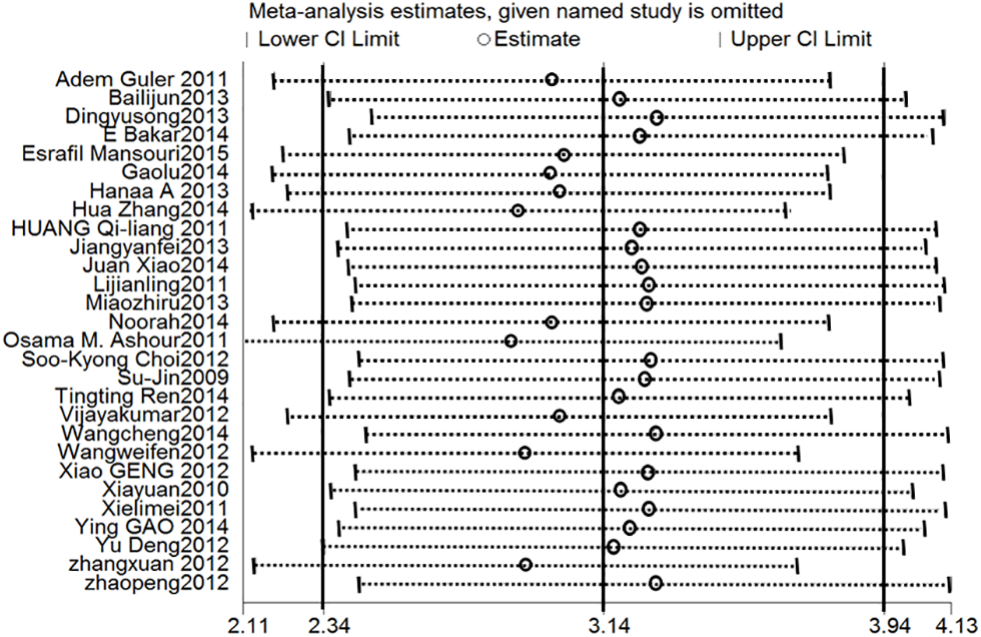
Sensitivity analysis for SOD. Stable results were observed for all studies, indicating that no individual study influenced the combined results. Abbreviations: SMD = standard mean difference, SE = standard error.

**Fig 10:**
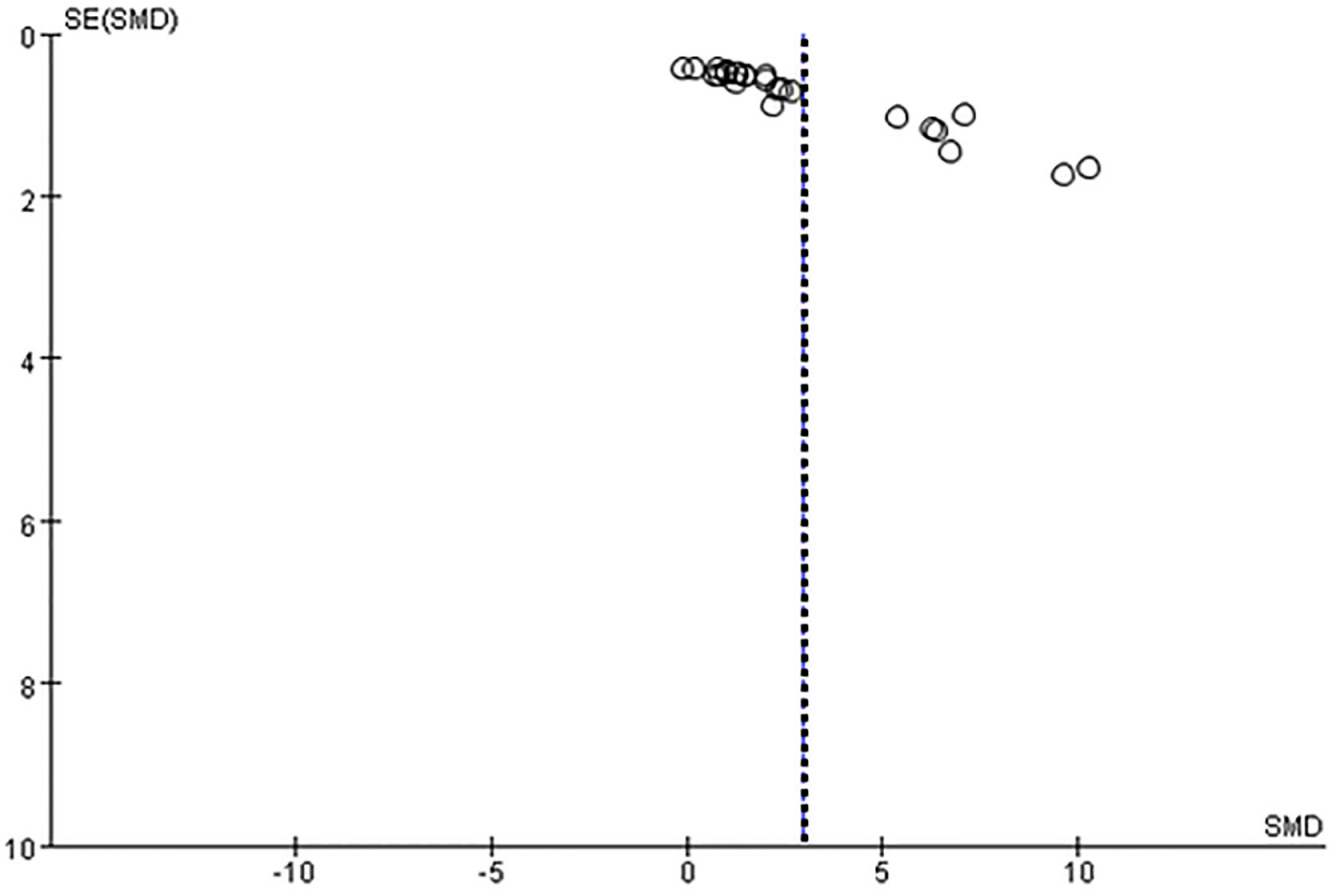
Funnel plot for the studies that estimated SOD. Dotted line shows the overall estimated standard mean difference.The figure showed that the studies distributed symmetrically around the overall mean estimate.

A sensitivity analysis was performed to evaluate the robustness of the study results. Specifically, we conducted a sensitivity analysis for SOD because it was estimated in 28 studies. Fig 9 shows the stability results for all studies; these results indicated that no individual study influenced the combined results. The method of intervention and the source of the outcome indicators (i.e., serum or tissue samples) significantly influenced the outcome indicators. A trend toward greater improvements was observed (Fig 8) when PC treatments were applied using the gavage method and when parameter estimates were based on tissue samples. The funnel plot for the studies that include estimates of SOD suggests that values were approximately evenly distributed around the overall mean estimate (Fig 10). Based on a multivariate meta-regression analysis, the source of outcome indicators (*P* = 0.040) and intervention method (*P* = 0.038) were significantly associated with differences in SOD.

## Discussion

Our results showed that PC intervention increases the levels of the antioxidative indicators SOD, CAT, GSH, GPx, and T-AOC, and decreases the concentration of MDA in oxidative damage mouse models. The reported effects of PCs were also influenced by other factors, such as the mode of intervention, treatment period, and sample source. Based on this meta-analysis of published papers, PCs have an obvious antioxidative effect.

PCs, a type of polyphenol, were first extracted from haw in Germany [43]. These compounds contain various amounts of catechin and epicatechin [44]. Depending on the degree of polymerization, dipolymer–tetramers are usually called oligomeric procyanidins, and others are usually called procyanidolic polymers [45]. The widely distributed dipolymers are the focus of research and are among the most important PCs [46].

PCs are excellent antioxidants and free-radical scavengers; their antioxidative ability exceeds that of vitamins C and E [47]. The results of this meta-analysis also indicated that PCs, which can effectively improve the activity of antioxidative enzymes and reduce lipid peroxidation products, have an obvious antioxidant effect. The influence of PCs on SOD, GPx, and CAT can be maximized by applying the gavage mode instead of the normal feeding mode. This may be attributed to the precise control of PC intake by the investigator when using the gavage method. PCs can more effectively enhance antioxidant enzyme activity in tissues than in serum. We speculated that the indicators in serum samples reflect the whole-body oxidation-antioxidation levels, rather than that of a specific organ or tissue. In addition, the effect of PCs on the T-AOC index was not significant, probably owing to the small sample size (i.e., 4 studies). The results of the subgroup analysis will facilitate the selection of detection indexes in future studies regarding the antioxidative effect of PCs.

**Fig 11:**
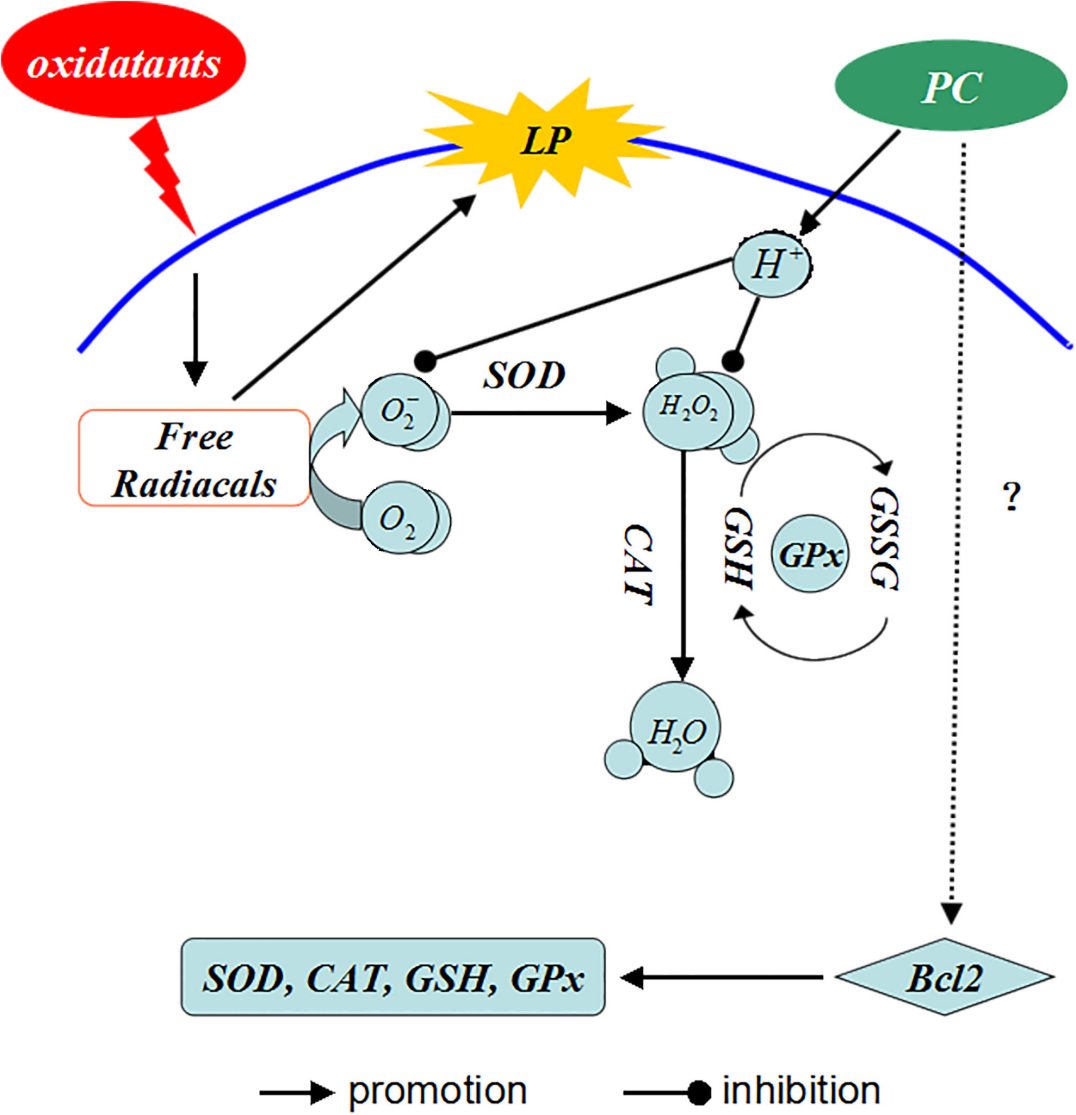
Antioxidant mechanism of PCs. PCs contain many phenolic hydroxyl groups and release H^+^ when they are oxidized, which can bind active oxygen radicals and competitively block the reaction chains of free radicals, reducing the consumption of antioxidants and increasing the activity of antioxidant enzymes. Abbreviations: LP represents lipid peroxidation, GSSG represents oxidized glutathione.

The antioxidative role of PCs is complex (Fig 11). Some harmful substances (such as H_2_0_2_, ethanol, galactose, and so on) induce oxidative stress and ROS production, and ultimately cause lipid peroxidation. The antioxidant defense system is activated and antioxidants (such as GSH, SOD, CAT, and GPx) remove excess free radicals and peroxides. If the degree of oxidation is beyond the capacity of antioxidant molecules, the levels of GSH, SOD, CAT, GPx, etc., will be reduced. PCs contain many phenolic hydroxyl groups and release H^+^ when they are oxidized, which can bind active oxygen radicals competitively to block the reaction chains of free radicals [48]. This reduces the consumption of antioxidants, increases the activity of antioxidative enzymes, improves antioxidative ability, and increases the T-AOC levels. In addition, it may be connected with increased expression of B-cell lymphoma-2 (Bcl-2), which can enhance antioxidation in cells. Liming [49], Yujie [50], and others have found that PC significantly increases the expression of Bcl-2, which increases the activity of antioxidant enzymes based on in vivo and vitro experiments.

This meta-analysis included 29 published papers. The quality of these studies was sufficient to analyze the combined effects of PCs. The sensitivity analysis demonstrated the robustness of the overall results. Similarly, the symmetric distribution of the studies in a funnel plot demonstrated the lack of a publication bias. Although there was heterogeneity among studies, the randomized effect model was used to integrate the results and a subgroup analysis and meta-regression were used to evaluate the heterogeneity. All of the above analyses support the validity of using the combined results of the 29 studies to determine the effect of PCs.

In summary, the results of the present study support the strong antioxidative effect of PCs as evidenced by the levels of oxidative stress indicators in a systematic review of relevant published papers. PCs are able to block the free radical chain reaction by eliminating radicals. They may also regulate the signaling pathway related to oxidative stress, thereby improving antioxidative activity. These results provide a scientific basis for the development and utilization of PC-based resources.

### Outlook

PCs have important antioxidative and antitumor effects and have protective effects with respect to the cardiovascular system and other biological activities [51]. They are widely used in medicine, health care products, and cosmetics [52]. Despite their wide use and increasing data related to their effects, the mechanisms that mediate the antioxidative effect of PCs are unclear. To improve product development and the utilization of PCs, it is necessary to determine the enzymes, receptor genes, and signaling pathways involved in the antioxidation process [53]. Additional examinations of the molecular mechanisms of PCs are needed to maximize their benefits with respect to human health.

### Limitations

A limitation of the present study was the obvious heterogeneity in the data. Heterogeneity was observed with respect to subgroup factors, animal strains, reagents, PC dosages, and many other factors. Using a funnel plot analysis, we detected some evidence for a publication bias. We only considered manuscripts published in English and Chinese in this study and were not able to retrieve negative results.

## Supporting Information

S1 File Raw Data of 29 studies. All data in the present study were extracted from 29 papers (references 14–42) and we display the data in the table as a supporting information to show the data availability.
(XLS)
